# Micro-expression recognition training in medical students: a pilot study

**DOI:** 10.1186/1472-6920-9-47

**Published:** 2009-07-20

**Authors:** Jennifer Endres, Anita Laidlaw

**Affiliations:** 1 Bute Medical School, University of St Andrews, Westburn Lane, St Andrews, Fife, UK

## Abstract

**Background:**

Patients provide emotional cues during consultations which may be verbal or non-verbal. Many studies focus on patient verbal cues as predictors of physicians' ability to recognize and address patient needs but this project focused on non-verbal cues in the form of facial micro-expressions. This pilot study investigated first year medical students' (n = 75) identified as being either good or poor communicators abilities to detect emotional micro-expressions before and after training using the Micro Expression Training Tool (METT) .

**Methods:**

The sample consisted of 24 first year medical students, 9 were from the lowest performance quartile in a communication skills OSCE (Objective Structured Clinical Exam) station and 15 were from the highest performance quartile. These students completed the METT individually, recording pre- and post-assessment scores. Students were also invited to provide their views on the training.

**Results:**

No difference in pre-assessment scores was found between the lowest and highest quartile groups (P = 0.797). After training, students in the high quartile showed significant improvement in the recognition of facial micro-expressions (P = 0.014). The lowest quartile students showed no improvement (P = 0.799).

**Conclusion:**

In conclusion, this pilot study showed there was no difference between the ability of medical undergraduate students assessed as being good communicators and those assessed as poor communicators to identify facial micro-expressions. But, the study did highlight that those students demonstrating good general clinical communication benefited from the training aspect of the METT, whereas low performing students did not gain. Why this should be the case is not clear and further investigation should be carried out to determine why lowest quartile students did not benefit.

## Background

Emotions, and their recognition in those we communicate with make it possible to behave flexibly in different situation as we regulate our social interactions[1]. One interaction where emotions are frequently shown by participants is the doctor-patient consultation. In his article 'Emotions revealed: recognising facial expressions' Paul Ekman states that recognising facial expressions, including the less obvious facial micro-expressions of patients may be useful to a doctor in their interactions [2]. Being able to perceive facial expressions accurately may aid in interpreting how much pain a patient is experiencing. In one study which interviewed Certified Nursing Assistants in an American care home one method the nursing assistants used to gauge the pain level in cognitively impaired residents was their facial expressions[3]. A further use would be to pick up clues to the patients emotional state. Archinard studied the behavioural responses of a doctor when interviewing patients who had attempted suicide[4]. Although the doctor appeared to pick up on facial expression cues from the patients to distinguish between those who would re-attempt suicide, as they behaved differently towards such patients; they were unable to use this information consciously to assign those patients as being at risk of re-attempting suicide. That is, although the doctor could discriminate and behave differently towards individuals who would repeatedly attempt suicide and those would not repeat, this information was not, or could not, be utilised when clinical decisions were made.

Emotional cues may be verbal or non-verbal[5]. Levinson et al found that responding to emotions expressed verbally by patients may result in shorter consultations[6], but the same study found that physicians responded positively to patients' verbal emotional cues in only 38% of surgery cases and 21% of primary care cases. Similar results were noted in oncologists in response to verbal cues from cancer patients, where only 28% of emotional cues were responded to appropriately[7]. Another study noted that cues were most likely to be missed by doctors if they did not directly state the emotional impact on the patient[8]. If a verbal message is ambiguous non-verbal behaviour, such as facial expression may elucidate what is meant[8,9].

There is mounting literature to suggest that a patient-centred model of care, whereby physicians address patients' emotional concerns *and *biomedical conditions should be adopted[10] and that such a positive interaction between doctors and patient is important for patient outcomes[11]. It is difficult to address emotional concerns if these are not recognised by doctors. Therefore the recognition of emotions in patients, using verbal or non-verbal cues is one of the important skills which can aid doctors in creating patient centred communications. Difficulties in communicating with patients have been shown in several studies to relate to complaints against doctors. For example, in a longitudinal study, Tamblyn et al. [12] reported that nearly one in five physicians had a retained complaint filed with the medical authorities in the first 2 to 12 years of practice, and physicians who scored in the lowest quartile of their Clinical Skills Exam (CSE) were at significantly greater risk of complaints than those in higher quartiles. Communication was one component of the CSE that was an important predictor of future complaints to the medical authorities.

One question this pilot study therefore wanted to ask was whether one reason for poor communication was due to an inability to recognise facial expressions. This was done by investigating whether there was a difference in the ability of medical students identified as good or poor communicators to perceive facial micro-expressions. Micro-expressions are brief (lasting up to 0.2 seconds) partial expressions which are less obvious than a full (or cardinal) facial expression[2]. The hypothesis tested was that individuals classified as good communicators would perceive facial micro-expressions more accurately than those classified as poor communicators. If this were indeed the case then this would provide us with one area that we could help such students in their clinical communication training.

Most medical schools currently incorporate an aspect of clinical communication training into their curriculum[12]. Training has been shown to be effective at improving the communicatory abilities of medical students, and these benefits can persist[13,14]. The training can employ a variety of methods including opportunities to practice particular skills with other students, or actors portraying the role of patients[15]. A 1989 paper by Lavelle[16] describes a course for medical students in 'The objective methods of clinical practice' a component of which was training in the recognition of full, cardinal, emotional facial expressions. Although no data is presented the author reports that 'Students' capacity to read single emotions remains much the same, but their ability to read multiple emotions improves dramatically'. In that study the students performance in the recognition of single full emotional facial expressions was maximal prior to training, whereas the recognition of multiple expressions was not and therefore training had an impact. A further question this pilot study wanted to investigate was whether skills such as recognition of facial micro-expressions could be taught explicitly to medical students.

Both research questions will be investigated by the use of the Micro-Expression Training Tool (METT) developed by Paul Ekman . The METT has been used previously to investigate the ability to perceive micro-expressions in a group of student participants[17] but there are currently no published studies investigating it's use in Health professionals.

## Methods

### Participants

Seventy-five pre-selected subjects consisting of first year medical students at the University of St. Andrews were invited to participate in this study. Pre-selection was based on the results of an OSCE (Objective Structured Clinical Exam) communications skills station half way through their first year of study, with those invited being in either the highest or lowest performance quartiles. The OSCE communication station involved talking to a simulated patient portraying a role in a General Practice context of someone who had fallen and hurt their ankle. The scoring tool contained both checklist and rating components. Checklist items included gathering information relating to the presenting complaint as well as past medical and social history. Areas where students were rated included introductions, ability to respond to patient perspective, concluding a consultation and a large proportion of the marks were available for global communication skills such as rapport/empathy, listening skills and non-verbal behaviour. Simulated patients also rated their satisfaction in the encounter. Students were recruited via an e-mail invitation to participate. Interested students were given an information sheet and a consent form.

### Materials and Procedures

During the study participants individually completed pre- and post-assessment, training, practice and review sections of the Micro-Expression Training Tool (METT)  under examination conditions. In the pre-assessment, subjects viewed fourteen flashed example faces of micro-expressions, consisting of either, disgust, sadness, happiness, contempt, fear, anger or surprise. Subjects were prompted to select one of the seven emotional labels. On completion of the pre-assessment a score, expressed as percentage correct, was assigned and recorded.

The next part of the CD ROM was a training session, where a narrator explained, in a slow-motion video clip, the four pairs of commonly confused emotions, e.g. fear/sadness, happy/contempt, surprise/fear and disgust/anger. The narrator provided explicit examples of differences and similarities in the regions of the eyes, nose and mouth. "The eyebrows are pulled down together in both these angry expressions . . . the biggest difference between them is in the mouth. The lips are pressed tightly on the right but they're open with tense lips, probably saying something quite unpleasant on the left". Following this was a practice session where subjects labelled 28 facial expressions, and, if incorrect, a still picture was paused for as long as necessary until the subject selected the correct emotional label. Like in the training section, the review used alternative faces to display four pairs of commonly confused expressions. The post-assessment followed the same procedure as the pre-assessment and again a score was recorded. Finally, participants were invited to provide open comments on any aspect of the METT and it's relation to their training in clinical communication.

Results were investigated for normality. All data apart from the OSCE results were normally distributed therefore the OSCE data was log transformed. Appropriate statistical tests (alpha level set to 0.05) were applied using SPSS v14.

This study was approved by the Bute Medical School Ethics Committee (MD3498).

## Results

### Quantitative results

Nine subjects from the lowest quartile (4 males, 5 females) and fifteen from the highest quartile (5 males, 10 females) consented to participate in the study (participation rate of 32%). The OSCE results of the students who volunteered to take part in the study were compared to the rest of the appropriate quartile cohort. There was no difference in OSCE scores between the volunteers and the rest of the cohort for either the lowest quartile (participant mean ± standard deviation = 42.19 ± 5.43, non-participant = 35.13 ± 14.82) or the highest quartile group (participant mean ± standard deviation = 81.25 ± 6.22, non-participant = 82.38 ± 4.80).

**Figure 1:**
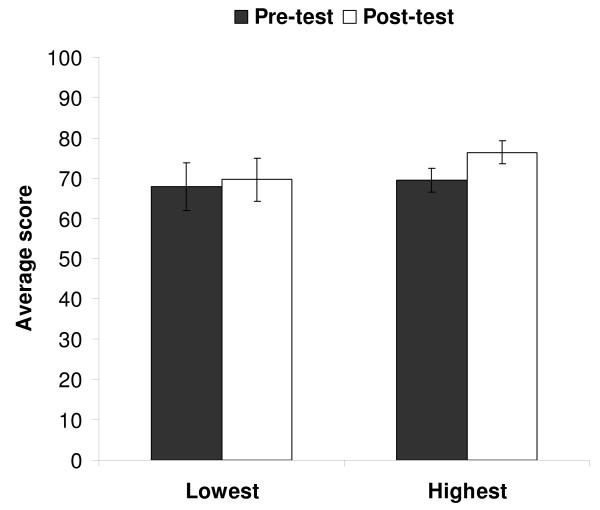
**Average scores for pre-and post-assessments**. Average scores on the pre- and post assessments of the METT for students in the lowest quartile and highest quartile groups. Error bars represent standard errors.

Surprisingly, there was no difference between the highest and lowest quartile students terms of METT pre-assessment scores (t = -0.261, df = 20, P = 0.797). When the difference between METT pre and post-assessment results were examined however a difference did emerge between the high and low quartile groups. No difference was found between the pre- and post-assessment score in the lowest quartile test subjects (paired t-test, t = -0.265, df = 7, P = 0.799), but there was a difference between these two scores for the highest quartile group (t = -2.580, df = 13, P = 0.014). Means and standard deviations of pre- and post-assessment scores are shown in Table 1. The highest quartile students improved their ability to identify facial micro-expression after training whilst the lowest quartile students did not (Figure 1).

**Table 1:** Mean and standard deviation of the pre- and post-assessments

		**Mean**	**Standard deviation**
**Lowest**	**Pre-assessment**	67.88	17.88
	**Post-assessment**	69.63	16.19

**Highest**	**Pre-assessment**	69.50	11.45
	**Post-assessment**	76.36	11.04

Mean and standard deviation of the pre and post training assessment scores for both the highest and lowest quartile groups.

### Qualitative comments

Students in both groups saw the relevance of the training. The following quotes were made by participants from the highest and lowest quartile groups respectively:

'I think this program was relevant and very insightful for those studying medicine. In an OSCE station this would come into use with patient-doctor consultation and history taking'

'Generally I thought this was a worthwhile study, and I particularly liked the training and the fact that it gave you the chance to recognise the actual expressions.'

They also found some aspects difficult, for example, one lowest quartile student commented:

'Some facial expressions were really hard to distinguish between, but the training really helped'.

Students however, saw some limitations in it's application, for example this student in the lowest quartile group:

'The training program was very good but the test showed the picture too quickly, so I thought it unrealistic. Especially when in the OSCE or real life I would be getting information from tone of voice and other body language'.

## Discussion

In the current study there was no difference between the abilities of students assessed as being either good or poor communicators in the METT baseline measure of perception of facial micro-expression (pre-assessment). This suggests that an inability to recognise facial expressions in patients was not the reason that these students were performing poorly in their communication skills assessments. There are various other potential reasons that communication with patients is ineffective including poor non-verbal communication behaviour from the health professional[18,19], or lack of appropriate verbal responses to cues from the health professional[20,21]. It may even be, as was suggested in the study by Archinard et al, that the facial expression information could be perceived by the health professional but not consciously acted upon[22]. From this small study it is impossible to determine which areas these students were poorly performing in.

The highest quartile students showed a significant improvement in their ability to perceive facial micro-expressions after training whilst the lowest quartile students did not, therefore the METT could be used to improve performance. Why there was a difference in improvement between the two groups in the current study is not clear, although it could be due to a variety of reasons including the low quartile group; requiring a longer period of training, having greater difficulty in perceiving the differences highlighted in the training, or being poorer at learning or less motivated to improve. Anxiety, including social anxiety may also impact on attention and learning[23,24]. This study could not be used to determine which of these possible reasons is valid for these students. Understanding why the higher quartile group benefited most is important for the potential to understand which aspects of the training improved their performance but did not impact on the lower quartile group and why this was the case. This could inform targeted training for future medical students. The students generally commented that they found training interesting and viewed it as useful.

This study has several limitations. The METT involves static facial micro-expressions. This may not be directly comparable to the ability to perceive such expressions in real time interactions, indeed this point was raised by one of the students in the study (see qualitative results). There are anatomical and physiological differences in brain response when an individual is viewing dynamic facial images compared to static images[25] and this may affect behavioural responses. Future work should concentrate on perception of facial expressions in video footage or real interactions.

Unfortunately the participation rate was low for this pilot study. More subjects would be required to confirm this effect and explore the link between assessed communication ability and improvement in perception of micro-expressions with training. This pilot study did however show the feasibility of utilising this CD ROM for undergraduate medical student training.

When considering facial expressions alone, Ekman[26] points out eight kinds to be aware of: from none to sub-visible, momentary, subtle, full, false, referential and mock. Our study was restricted to momentary facial micro-expressions, therefore the ability to perceive other types of expressions was not investigated. Previous work has shown that medical students do benefit from training in the recognition of multiple facial expressions but not full facial expressions[16]. The present study therefore adds to this knowledge with the more subtle facial micro-expression.

There is also a lack of evidence of benefit to communication behaviour in this study. The ethos of breaking down communication into micro-skills is the basis for one of the most frequently used guides for clinical communication skills training in undergraduate medical education, the Calgary Cambridge Guides[27]. This project is an attempt to understand the cognitive basis for one of the micro-skills involved in communication, micro-expression perception, and the ability to improve that micro-skill through training.

## Conclusion

This pilot study provides initial evidence that after training in the recognition of static facial micro-expressions medical students identified as poor communicators do not improve whilst those identified as good at communication do. This finding should be further explored to understand the basis of this difference and how best to target training for future medical undergraduates. Further, the impact of such training on clinical communication should be assessed.

## Competing interests

The authors declare that they have no competing interests, including no connections with the Paul Ekman Group.

## Authors' contributions

JE organized and ran the study and drafted an early version of this manuscript. AL made substantial contributions to the concept and design of the study and revised this manuscript. All authors read an approved the final manuscript.

## Pre-publication history

The pre-publication history for this paper can be accessed here:


